# Serum microRNA profiling for the identification of predictive molecular markers of the response to controlled ovarian stimulation

**DOI:** 10.5935/1518-0557.20190070

**Published:** 2020

**Authors:** Edson Borges Jr., Maria Gabriela Ferreira Mulato, Amanda Souza Setti, Assumpto Iaconelli Jr., Murilo Vieira Geraldo, Daniela Paes de Almeida Ferreira Braga

**Affiliations:** 1 Fertility Medical Group; 2 Departamento de Biologia Estrutural e Funcional, Instituto de Biologia, Universidade Estadual de Campinas - UNICAMP; 3 Instituto Sapientiae – Centro de Estudos e Pesquisa em Reprodução Assistida

**Keywords:** poor responder, hyper responder, COS, biomarker, microRNA, stimulation

## Abstract

**Objective:**

To identify potential microRNA (miRNA) biomarkers of poor, normal and hyperresponse to controlled ovarian stimulation (COS).

**Methods:**

In the present study, we assessed 40 serum samples from patients undergoing COS. We used ten samples to standardize miRNAs detection in the serum. The remaining 30 samples were split into three groups depending on the patient's response to COS: poor response (PR group, n=10), normal response (NR group, n=10), and hyperresponse (HR group, n=10). Aberrantly expressed miRNAs were identified using a large-scale expression analysis platform. Gene set enrichment analysis was performed to assess the biological processes potentially modulated by the identified miRNAs.

**Results:**

Twenty-two miRNAs were detected only in the PR or HR groups when compared with the NR group. From those, 11 presented poor dissociation curves and were excluded from further analysis. A bioinformatics analysis revealed that the selected 11 miRNAs target several genes involved in GnRH, estrogen and prolactin signaling, oocyte maturation, female pregnancy, and meiosis.

**Conclusion:**

The large-scale analysis of miRNA expression identified distinct miRNA profiles for poor and hyperresponse to COS, which potentially modulate key processes for human assisted reproduction. All evidence suggests that the serum microRNA profiling may discriminate patients who will respond in an exacerbated manner from those who will respond insufficiently to COS. Further studies may validate these miRNAs, enabling the individualization of treatment and more successful outcomes.

## INTRODUCTION

Since the first reported pregnancy following *in vitro* fertilization (IVF) (Steptoe & Edwards, 1978), assisted reproductive technologies (ART) have quickly progressed. It is estimated that over 237,000 infants are born through ART worldwide in a single year (Sullivan *et al*., 2013). Nevertheless, its efficiency in terms of live births is still low. It has been estimated that 4.45 cycles are needed to achieve one live birth event following IVF (Sunkara *et al*., 2016). Many variables have been suggested as possible causes for the low efficiency of assisted reproduction. Embryo implantation depends on the acquisition of a receptive endometrium and the presence of a viable embryo (Su & Fazleabas, 2015). The selection of a viable embryo for transfer is a critical step in ART and depends on an adequate number and quality of oocytes. However, the response to controlled ovarian stimulation (COS) is quite variable among patients, and both poor and hyperresponses may have detrimental effects on the cycle's outcome.

Previous registers have reported important data concerning the cancellation rates of IVF cycles (Sullivan *et al*., 2013; Sunkara *et al*., 2016). We could assumed that, for young women, a significant number of cycles are likely to be cancelled because of the risk of ovarian hyperstimulation syndrome (OHSS), rather than because of a poor ovarian response. On the other hand, for older women, cycle cancellation is more likely to be associated with a poor ovarian response.

According with the consensus from the European Society of Human Reproduction an Embryology (ESHRE), a poor ovarian response is defined as the collection of fewer than four oocytes in response to a COS protocol of at least 150 IU FSH per day (Ferraretti *et al*., 2011). The management of patients with poor ovarian response to exogenous gonadotropin stimulation has challenged reproductive specialists for decades. There is a great need to attain an optimal oocyte yield while minimizing the risk of an excessive response and OHSS (Nyboe Andersen *et al*., 2017).

Although OHSS is a condition with a small risk of mortality, it is an important condition with considerable morbidity (Corbett *et al*., 2014; Boothroyd *et al*., 2015), and it remains the most critical safety concern associated with the use of gonadotropin preparations. Previous reports have shown a positive linear relationship between oocyte yield and live birth rates of up to 15 to 25 oocytes, using conventional ovarian stimulation (Sunkara *et al*., 2011; Polyzos *et al*., 2018). Beyond 15 oocytes, there is no increase in live birth rates, but the risk of OHSS increases exponentially (Sunkara *et al*., 2011; Steward *et al*., 2014). Within this line, it is crucial to implement less aggressive stimulation while maintaining an adequate oocyte yield.

Age, serum FSH, antral follicle count (AFC), serum AMH, and other factors have been pointed out as possible predictive factors to ovarian response to COS. However, there is no consensus regarding which factors to take into account or the importance of each factor when determining the dose of gonadotrophins for COS (Corbett *et al*., 2014; Boothroyd *et al.*, 2015; Oudshoorn *et al*., 2017; van Tilborg *et al*., 2017a; 2017b; Lensen *et al*., 2018). In addition, it is nearly impossible to accurately predict the ovarian response and tailor an individualized stimulation protocol based on such parameters. Thus, the development of non-invasive techniques, capable of predicting the response to COS, would allow for individualized treatment and significantly increase treatment success while decreasing the physical, emotional and economic burden on patients.

MicroRNAs (miRNAs) are endogenous, evolutionally conserved, single-strand non-coding RNA molecules of 20-24 nucleotides that post-transcriptionally regulate gene expression in eukaryotes, including mammalian cells (Asirvatham *et al*., 2009; McCallie *et al*., 2010; Mouillet *et al*., 2015; Thouas *et al*., 2015). They were first described in the nematode *Caenorhabditis elegans* (Lee *et al*., 1993; Wightman *et al*., 1993) and later found in the genomes of protists, plants, animals, and viruses (Mouillet *et al*., 2015). In mammals, it has been shown that miRNAs are actively secreted into the bloodstream and regulate gene expression in distant organs (Thomou *et al*., 2017a; 2017b). In fact, miRNAs have been detected in virtually all human bodily fluids, including blood, urine, saliva, tears, breast milk, semen, amniotic fluid, cerebrospinal fluid, peritoneal fluid, and pleural fluid as well as in culture media collected from different cell lines (Wang *et al*., 2010 a; 2010b; Weber *et al*., 2010).

The study of signaling molecules, such as miRNAs, in the serum of patients undergoing IVF may be a valuable approach to predict the response to COS and facilitate the development of an individualized gonadotrophin dosing strategy. Therefore, the goal of the present study was to identify potential miRNAs biomarkers of poor, normal, and hyperresponse to COS.

## MATERIALS AND METHODS

### Study Design

For the present study, we analyzed 40 serum samples derived from patients undergoing COS for intracytoplasmic sperm injection (ICSI). The samples were collected in a private university-affiliated IVF center, between January 2017 and January 2018.

We used ten samples to standardize the detection of serum miRNAs. The remaining 30 samples were split into three groups depending on the patient's response to COS: poor response (PR group, n=10), normal response (NR group, n=10), and hyperresponse (HR group, n=10). The aberrantly expressed miRNAs were identified using a large-scale expression analysis platform. In addition, we carried out a gene set enrichment analysis to access the biological processes potentially modulated by the identified miRNAs.

The study included only patients undergoing their first or second IVF cycle, using fresh oocytes and/or embryos, and aged <38 years old. We obtained a written informed consent, in which patients agreed to share the outcomes of their cycles for research purposes, and the local institutional review board approved the study.

### Sample collection

Serum samples were obtained through venipuncture, prior to beginning the COS. After complete filling of a serum separation tube, the blood was carefully mixed with coagulation activating agent and remained standing for 60 minutes. After complete coagulation, the samples were centrifuged at 1500 to 2000xg for 10 minutes until the serum was well separated. Using a Pasteur pipette, the serum was transferred to another tube and stored at -20°C.

### Controlled ovarian stimulation and laboratory procedures

Controlled ovarian stimulation was performed using recombinant FSH (Gonal-F^®^, Merck KGaA, Darmstadt, Germany) and pituitary suppression using a GnRH antagonist (GnRH - Cetrotide^®^ Merck KGaA, Darmstadt, Germany). Follicular growth was monitored using transvaginal ultrasound starting on day 4 of the gonadotropin administration. When adequate follicular growth and serum E2 levels were observed, recombinant hCG (Ovidrel^®^, Merck KGaA, Darmstadt, Germany) or leuprolide acetate (Lupron^®^, AbbVie, Boston, USA) was administered to trigger the final follicular maturation. The oocytes were collected 35 hours after hCG administration through transvaginal ultrasound ovum pick-up.

The recovered oocytes were assessed to determine their nuclear status, and those in metaphase II were submitted to ICSI following routine procedures (Palermo *et al.,* 1997).

### MiRNA extraction, identification and quantification

A small RNA fraction was extracted from 200µl of serum samples, using a miRNeasy Serum/Plasma extraction kit (QIAGEN, Hilden, Germany). *C. elegans miR-39* was used as an RNA spike-in to normalize the gene expression analysis (miRNeasy Serum/Plasma Spike-in Control, QIAGEN, Hilden, Germany).

The identification and quantification of aberrantly expressed miRNAs, in the pool of samples form PR, NR and HR groups, was performed by using a large-scale quantification PCR-Array platform (miScript^®^ QIAGEN, Helden, Germany) on a ViiA7 apparatus (Thermo-Fisher, Massachusetts, USA). We analyzed the data using a PCR Array Data Analysis Web Portal software (http://pcrdataanalysis.sabiosciences.com/mirna).

### Gene set enrichment analysis

The list of putative targets for the selected miRNAs for each group (HR or PR) was obtained using the miRWalk 2.0 web tool (http://zmf.umm.uni-heidelberg.de/apps/zmf/mirwalk2/). For each group, the lists of predicted targets for each miRNA were combined, and those targets predicted by 7 out of the 12 algorithms, or less, were excluded. The remaining target list was then submitted to enrichment analysis using the DAVID Annotation tool (https://david.ncifcrf.gov/).

### Statistical analyses

Patient and cycle characteristics were analyzed using the SPSS Statistics 21 (IBM, New York, NY, USA) statistical program. The variables were tested for normality distribution and group homogeneity using the Shapiro Wilk and Levene tests, respectively. When necessary, the samples were normalized using z-scores. The groups were compared by ANOVA, followed by the Bonferroni post-hoc test. Variables are described as mean ± standard deviation and the significance level α was set at 5%.

## RESULTS

Table 1 depicts the characteristics of the patients and cycles. As expected, the level of estradiol on trigger day, the number of aspirated follicles, the number of retrieved follicles, and the number of mature follicles were higher for the HR group, followed by the NR group, while the PR group presented the lowest results.

**Table 1:** Patient and cycle characteristics for the poor response, normal response and hyperresponse groups for the first experimental set

	PR(n=5)	NR(n=5)	HR(n=5)	*p*
Age (years)	33.88±1.87	32.40±2.75	31.30±2.11	0.065
BMI (kg/m^2^)	25.04±4.27	22.44±2.50	25.50±4.19	0.264
FSH dose (IU)	2383.33±668.48	2550.00±469.87	2495.00±584.38	0.825
Estradiol level (pg/ml)	913.00±415.80^a^	1818.00±1073.19^b^	3901.00±770.74^c^	*0.003*
Aspirated follicles (n)	3.50±0.85^a^	11.20±1.03^b^	41.40±22.24^c^	<*0.001*
Retrieved oocytes (n)	2.80±0.92^a^	9.70±1.49^b^	28.50±4.45^c^	<*0.001*
Oocyte retrieval rate (%)	82.50±23.71	86.82±11.84	81.03±24.98	0.816
Mature oocytes (n)	2.40±0.84^a^	7.30±1.56^b^	23.80±5.09^c^	<*0.001*

PR=poor response, NR=normal response, HR=hyperresponse, BMI=body mass index. a ≠ b ≠ c (one-way ANOVA followed by Bonferroni Post hoc test, *p*<0.05)

After the large-scale analysis of miRNA, we identified 22 miRNAs with amplification exclusively in the PR or HR groups when compared with the NR group. From the 22 miRNAs, 11 had poor dissociation curves, probably due to non-specific amplifications or primer dimer amplifications. Therefore, those products were excluded from further analysis (Table 2). Of the remaining 11 miRNAs, *miR-99a-5p, miR-181d-5p, miR-221-3p, miR-92a-1-5p,* and *miR-1302* were only detected in the HR groups, while *miR-150-5p, miR-223-3p, let-7d-3p, miR-891a-5p, miR-99a-3p* and*, miR-200c-5p* were only detected in the PR group (Table 2).

**Table 2:** miRNAs differentially detected in the serum of patients to be submitted to COS for ART cycles.

miRNA ID	CT value			Fold Change	
	**NR**	**PR**	**HR**	**PR *vs.* NR**	**HR *vs.* NR**
*hsa-miR-99a-5p*	n.d.	n.d.	36.661	-	8.60
*hsa-miR-181d-5p*	n.d.	n.d.	31.444	-	52.99
*hsa-miR-221-3p*	n.d.	n.d.	35.203	-	3.91
*hsa-miR-92a-1-5p*	n.d.	n.d.	34.994	-	4.52
*hsa-miR-1302*	n.d.	n.d.	34.614	-	21.67
*hsa-miR-150-5p*	n.d.	35.811	n.d.	13.60	-
* hsa-miR-223-3p*	n.d.	35.933	n.d.	16.76	-
*hsa-let-7d-3p*	n.d.	36.662	n.d.	10.11	-
*hsa-miR-891a-5p*	n.d.	34.248	n.d.	40.1	-
*hsa-miR-99a-3p*	n.d.	36.335	n.d.	9.01	-
*hsa-miR-200c-5p*	n.d.	34.829	n.d.	36.03	-

n.d.=not detectable; Ct value=cycle threshold value, cycle where fluorescence level exceeds detection threshold; NR=normal response; PR=poor response; HR=hyperresponse.

**Figure 1:**
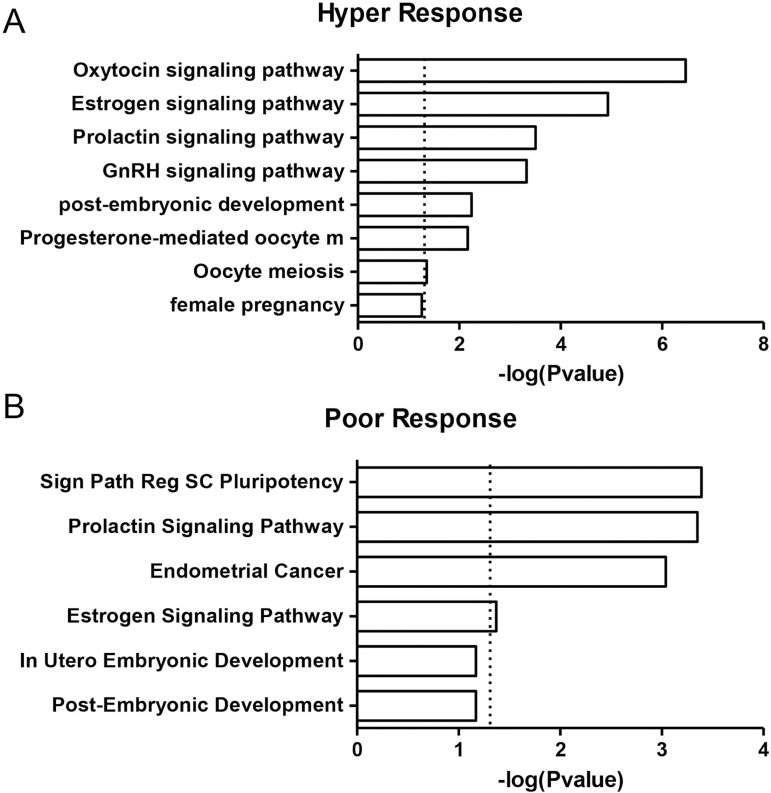
Gene set enrichment analysis of differentially detected miRNAs in HR and PR groups. The bars represent biological processes and signaling pathways enriched among the predicted target genes of the miRNAs from HR and PR groups. Values on the x-axis are represented as -log *p* values.

**Figure 2:**
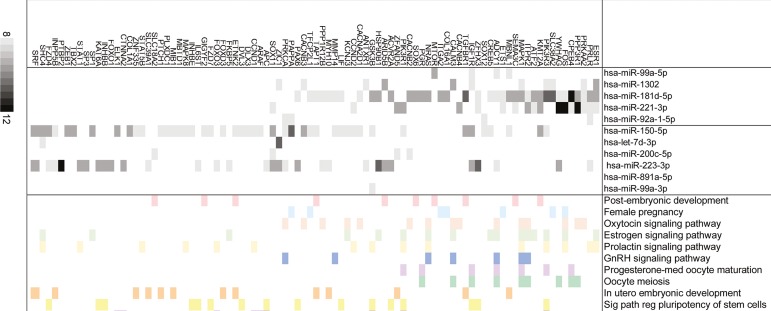
miRNAs from HR and PR groups and their target genes. Representative list of miRNA targets and enriched processes. The grey boxes indicate the number of prediction algorithms that consider that interaction possible (from 8 to 12) using the miRWalk web tool. Colored boxes indicate that each target gene is annotated in the given ontological category according to the DAVID platform.

The lists of predicted targets for each miRNA in both HR and PR groups were obtained and the gene set enrichment analysis (GSEA) was performed. The GSEA revealed that miRNAs from the HR group potentially modulate key members of GnRH, estrogen, prolactin, and oxytocin signaling pathways, as well as progesterone-mediated oocyte maturation and female pregnancy (Figures 1 A and 2). It is worth highlighting that *miR-181d-5p* targets comprised more than 40% of the targets in the HR group. On the other hand, miRNAs from the PR group potentially modulate members of the prolactin and estrogen signaling pathways in utero, embryo and post-embryonic development, and the genes involved in stem cell pluripotency and endometrial cancer (Figures 1 B and 2). *miR-150-5p* and *miR-223-3p* represent more than 49% and 32% of the targets, respectively.

## DISCUSSION

The main objective of treatment individualization in IVF is to offer every single woman the best treatment tailored to her unique characteristics, thus maximizing success, eliminating iatrogenic risks such as OHSS, and minimizing the risk of cycle cancellation. However, the success of individualized COS depends on finding a reliable method for predicting the ovarian response to stimulation.

For the present study, potential miRNA biomarkers of the response to ovarian stimulation were identified in the serum of patients undergoing ART, before COS. Extensive research has been carried out in a search for markers that can be used to facilitate COS individualization. Previous studies have focused on the assessment of miRNAs as molecular markers in assisted reproduction. It has been found that a poor response to COS is associated with altered miRNA expression, specifically with elevated *miR-21-5p* expression (Karakaya *et al*., 2015) or miRNA-15a-5p (Zhang *et al*., 2017). These studies, however, analysed the exression on miRNAs in granulosa cells, which can only be retrived during ovum pick-up, which limits the clinical usefulness of these biomarkers for the management of COS.

In this manner, the study of altered expression of miRNAs in serum may be more useful. Zhao *et al*. (2015) evaluated the expression of serum miRNAs and their predictive value for OHSS in patients with polycystic ovarian syndrome (PCOS). Two miRNAs were differently expressed in PCOS patients likely to suffer from severe OHSS. The value of the aforementioned article is indisputable, since it is well-known that PCOS patients who undergo COS are at a high risk of OHSS. However, for the application of an individualised stimulus treatment, other patient categories should also be investigated.

In our study, we aimed at identifying miRNAs in the serum of patients with different responses to COS. Several miRNAs, expressed only in poor and hyperresponders, were identified in a large-scale analysis by the qPCR array platform. miRNAs analyses of target genes indicated that these molecules are key player candidates. Moreover, the levels of this miRNA positively correlate with important clinical aspects, such as the number of retrieved oocytes.

Altered expression of *miR-181d-5p* has been described in various types of cancer, including ovarian carcinoma (Lee *et al*., 2012; Neel & Lebrun, 2013; Pal *et al*., 2015; Mahdian-Shakib *et al*., 2016; Widodo *et al*., 2016). It is also known that the regulation of this miRNA expression in endometrial stromal cells plays a role in the decidualisation process (Estella *et al*., 2012). In addition, miR-181d-5p serum levels are predictive of non-alcoholic fatty liver disease, and can be used in the differential diagnosis between autoimmune pancreatitis and pancreatic carcinoma (Celikbilek *et al*., 2014; Akamatsu *et al*., 2016). However, the biological function of *miR-181d-5p* in ovarian physiology is still to be unveiled.

A study by Khan *et al*. (2015) suggests that *miR-150* expression, detected only in the PR group, is decreased during ovarian development in mice. In addition, it has been shown that *miR-150* is expressed in higher levels in atretic rather than healthy follicles in cattle, and may play a role in bovine oocyte maturation (Donadeu *et al*., 2017). Importantly, *miR-150* expression is decreased in patients with endometriosis treated with simvastatin (Cosar *et al*., 2018).

Thus, miRNA biomarkers continue to emerge as potential tools for the management of infertility. Future analyses for the detection of these miRNAS in a large number of samples could contribute to their validation as diagnostic tools for the prediction of ovarian response to COS.
